# Draft genome sequence of three hydrocarbon-degrading *Pseudomonadota* strains isolated from an abandoned century-old oil exploration well

**DOI:** 10.1128/mra.01076-23

**Published:** 2024-01-30

**Authors:** Jefferson Camacho, Esteve Mesén-Porras, Diego Rojas-Gätjens, Danilo Pérez-Pantoja, Fernando Puente-Sánchez, Max Chavarría

**Affiliations:** 1 Centro Nacional de Innovaciones Biotecnológicas (CENIBiot), CeNAT-CONARE, San José, Costa Rica; 2 Escuela de Química, Universidad de Costa Rica, San José, Costa Rica; 3 Instituto Universitario de Investigación y Desarrollo Tecnológico, Universidad Tecnológica Metropolitana, Santiago, Chile; 4 Center of Applied Ecology and Sustainability (CAPES), Santiago, Chile; 5 Department of Aquatic Sciences and Assessment, Swedish University of Agricultural Sciences, Lennart Hjelms väg, Uppsala, Sweden; 6 Centro de Investigaciones en Productos Naturales (CIPRONA), Universidad de Costa Rica, San José, Costa Rica; The University of Arizona, USA

**Keywords:** hydrocarbon-degrading bacteria, *Pseudomonadota*, *Pseudomonas*, *Acinetobacter*, oil exploration well, Cahuita

## Abstract

We present genome sequences of three *Pseudomonadota* strains isolated from an abandoned century-old oil exploration well. A *Pseudomonas* sp. genome showed a size of 5,378,420 bp, while *Acinetobacter* genomes sized 3,522,593 and 3,864,311 bp. Genomes included catabolic genes for benzoate, 4-hydroxybenzoate, salicylate, vanillate, indoleacetate, anthranilate, *n*-alkanes, 4-hydroxyphenylacetate, phenylacetate, among others.

## ANNOUNCEMENT

Studies focused in hydrocarbon bioremediation have highlighted the potential of exploring microorganisms containing genes related to hydrocarbon-degrading enzymes (1, 2). Specifically, bacteria belonging to genera *Pseudomonas* (3) and *Acinetobacter* (4) have shown potential to degrade alkanes and aromatic hydrocarbons (5, 6). Here, we report the genomic sequencing of three strains retrieved from an abandoned old-century oil exploration well, located in the Cahuita National Park, Costa Rica, which is characterized by a continuous efflux of methane and the presence of a complex mixture of hydrocarbons (7). Sampling details and isolation methods are described by Rojas-Gätjens et al. (7). Such microorganisms were identified as one strain of *Pseudomonas* sp. termed C11 and two different *Acinetobacter* strains termed C4 and C10.

Isolates stored in glycerol 20% at –80°C were grown in lysogeny broth (8) (150 mL) and incubated overnight (180 rpm, 30°C). Genomic DNA extraction was performed using cetyltrimethylammonium bromide protocol (9). DNA was sequenced at Novogene company Ltd., Singapore. A paired-end sequencing library was prepared using the Illumina HiSeq Preparation Kit and loaded onto NovaSeq PE150 system. The raw data quality was evaluated using FastQC v0.11.9 (10), and low-quality reads and adapter sequences were filtered using Trimmomatic v0.36 (11). The remaining reads were assembled with SPADES v5.2.0 (12), followed by assembly polishing through Pilon v1.24 (13). Contigs smaller than 500 bp were removed with BBMap suite v37.36 (14). The quality assessment of each assembly was summarized using QUAST v5.2.0 (15). Genome annotation was determined using NCBI Prokaryotic Genome Annotation Pipeline (16). Default parameters were used for all software. Genome assembly statistics and annotation features are presented in Table 1.

**TABLE 1:** Annotation and genome assembly statistics of three hydrocarbon-degrading strains belonging to *Pseudomonas* and *Acinetobacter* genera isolated from an abandoned century-old oil exploration well

Isolate Feature	*Pseudomonas* sp. C11	*Acinetobacter johnsonii* C4	*Acinetobacter pittii* C10
BioProject accession no.	PRJNA997083	PRJNA997083	PRJNA997083
Assembly accession no.	JAWIIP000000000.1	JAVQMB000000000.1	JAWIIO000000000.1
No. of raw reads	1,175,0304	7,211,368	7,795,620
Genome size (bp)	5,378,420	3,522,593	3,864,311
No. of contigs	35	59	14
No. of predicted genes	4,943	2,009	1,812
Coverage (1×) (bp)	51,813	78,898	72,299
G + C content (%)	62.85	41.44	38.72
*N*_50_ (bp)	312,100	113,976	2,118,397
No. of tRNA	60	63	61
No. of rRNA	3	3	3
No. of ncRNA	33	11	30

**Fig 1:**
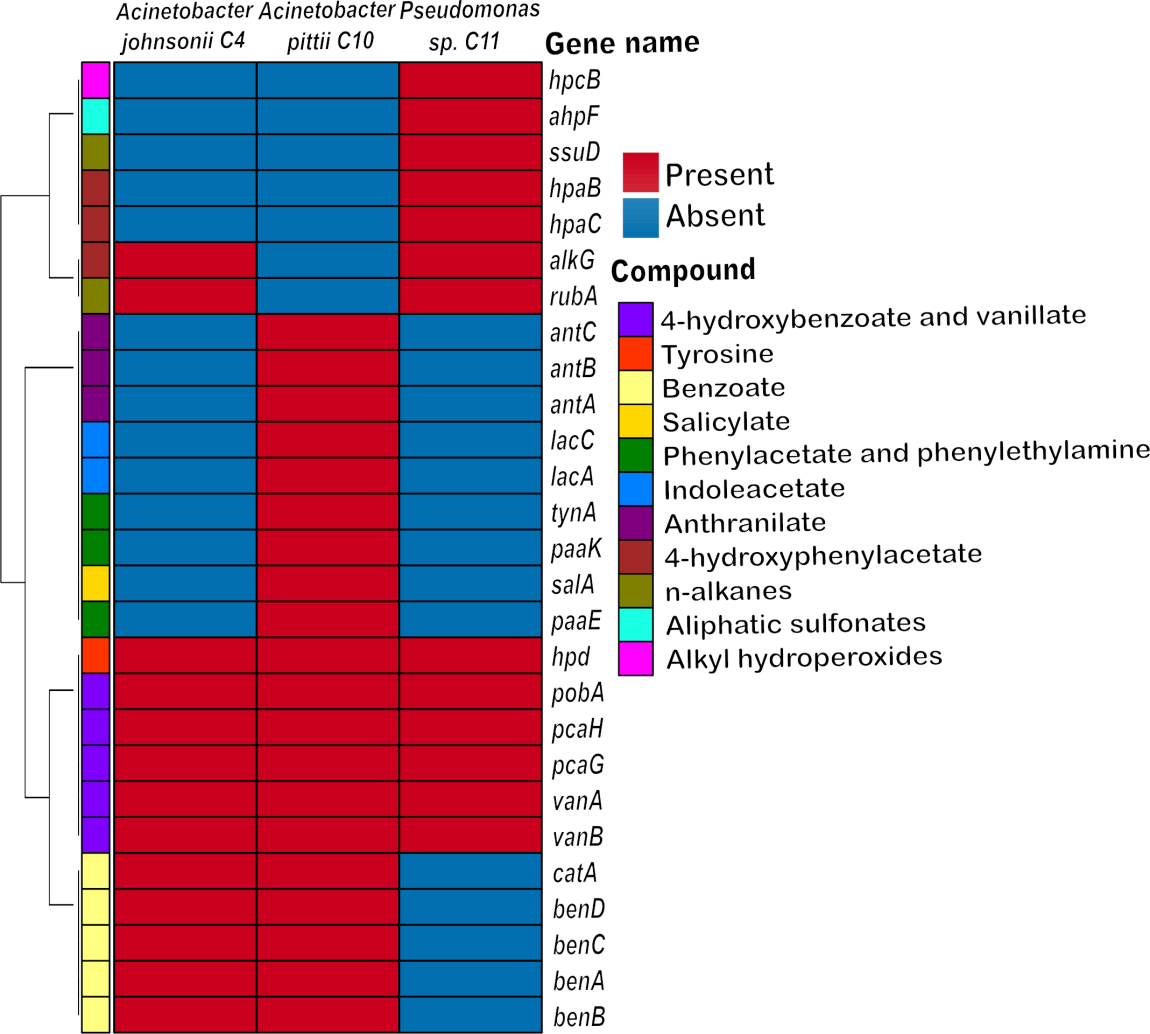
Genes related to hydrocarbons biodegradation identified in three bacterial strains isolated from an abandoned century-old oil exploration well in Cahuita National Park, Costa Rica. The color scale in the heatmap indicates the absence (light blue color) or presence (reddish color) of 27 different catabolic genes related to the biodegradation of specific aromatic or aliphatic hydrocarbons. The gene names are indicated on the right of the heatmap. The dendrogram on the left represents the clustering of the genes based on the Manhattan algorithm. Gene description of the protein product: *hpcB*, homoprotocatechuate 2,3-dioxygenase; *ahpF*, alkyl hydroperoxide reductase subunit F; *ssuD*, alkanesulfonate monooxygenase; *hpaB*, 4-hydroxyphenylacetate 3-monooxygenase, oxygenase component; *hpaC*, 4-hydroxyphenylacetate 3-monooxygenase, reductase component; *alkG*, rubredoxin, subunit 2; *rubA*, rubredoxin, subunit 1; *antC*, anthranilate 1,2-dioxygenase, reductase component; *antB*, anthranilate 1,2-dioxygenase, small subunit of terminal oxygenase component; *antA*, anthranilate 1,2-dioxygenase, large subunit of terminal oxygenase component; *lacC*, laccase domain-containing protein 1; *lacA*, galactoside acetyltransferase; *tynA*, 2-phenylethylamine oxidase; *paaK*, phenylacetate-coenzyme A ligase; *salA*, salicylate 1-hydroxylase; *paaE*, 1,2-phenylacetyl-CoA epoxidase, subunit E; *hpd*, 4-hydroxyphenylpyruvate dioxygenase; *pobA*, phenoxybenzoate dioxygenase, alpha subunit; *pcaH*, protocatechuate 3,4, dioxygenase, beta subunit; *pcaG*, protocatechuate 3,4, dioxygenase, alpha subunit; *vanA*, vanillate O-demethylase, oxygenase subunit; *vanB*, vanillate O-demethylase, oxidoreductase subunit; *catA*, catechol 1,2-dioxygenase; *benD*, 1,6-dihydroxycyclohexa-2,4-diene-1-carboxylate dehydrogenase; *benC*, benzoate 1,2-dioxygenase, electron transfer component; *benA*, benzoate 1,2-dioxygenase, alpha subunit; *benB*, benzoate 1,2-dioxygenase, beta subunit.

Taxonomic analysis was performed using the tetra correlation search tool of JspeciesWS v4.0.2 (17). This analysis showed that C4 strain is related to *Acinetobacter johnsonii*, C10 is closely related to *Acinetobacter pittii,* and C11 only can be classified as *Pseudomonas* sp. Consistently, average nucleotide identity (18) showed that C4 and C10 share more than 96% and 99% of its genome with *A. johnsonii* and *A. pittii* strains, respectively. Nonetheless, there were no identity values above the species threshold (>95%) for C11, suggesting that such isolate could represent a new *Pseudomonas* species. To evaluate the metabolic capacities of each strain, we analyzed the proteome generated by genome annotation to identify enzymes related to hydrocarbons biodegradation by using the Hydrocarbon Aerobic Degradation Enzymes and Genes database (19) and the ProteinOrtho v6.3.0 tool (20). A matrix for plotting a heatmap was built using tidyverse (21), RColorBrewer v1.1–3 (22), colorRamps v2.3.1, and ComplexHeatmap v2.14.0 (23). The results are showed in Fig. 1, revealing the presence of genes related to aromatic compounds degradation, including benzoate, 4-hydroxybenzoate, vanillate, salicylate, anthranilate, indoleacetate, and phenylacetate as well medium- and long-chain alkanes.

## Data Availability

The whole-genome sequencing shotgun for the three microorganisms was submitted to NCBI/GenBank under BioProject PRJNA997083. Raw reads were deposited with SRA numbers SRR25386955, SRR25386957, SRR25386958 and the assembled genomes with accessions JAWIIP000000000.1, JAWIIO000000000.1, JAVQMB000000000.1. The current version referred to this paper is identified as version 1.0.
